# Sutureless technique using the left atrial appendage for left pulmonary vein occlusion after catheter ablation

**DOI:** 10.1186/s40792-020-00930-w

**Published:** 2020-07-23

**Authors:** Naohiro Wakabayashi, Aina Hirofuji, Natsuya Ishikawa, Hiroyuki Kamiya

**Affiliations:** Department of Cardiac Surgery, Asahikawa Medical University, Midorigaoka Higashi 2-1-1-1, Asahikawa, 078-8510 Japan

**Keywords:** Sutureless technique, Pulmonary vein stenosis, Catheter ablation

## Abstract

**Background:**

Pulmonary vein stenosis or occlusion is a rare but one of the most devastating complications after catheter ablation for cardiac arrhythmias, and surgical repair is an option in severe cases. The sutureless technique, which avoids direct suture of vessel walls, was initially described for congenital pulmonary vein stenosis and has been widely performed due to its good restenosis-free rate.

**Case presentation:**

A 52-year-old male developed left pulmonary vein occlusion after catheter ablation for atrial fibrillation. The surgical repair with sutureless technique using the left atrial appendage was performed without any complications. Postoperative computed tomography demonstrated the revascularization of the pulmonary vein.

**Conclusions:**

The sutureless technique using the left atrial appendage is significantly reasonable particularly in case of left pulmonary vein stenosis or occlusion after catheter ablation for atrial fibrillation since it reduces the risks of restenosis and thromboembolism.

## Background

Pulmonary vein (PV) stenosis occurs as a complication of catheter ablation for cardiac arrhythmia in 1–3% of patients [1]. Percutaneous interventions such as balloon angioplasty or stenting are initially performed; however, since they are still challenging procedures with some complications such as PV perforation or pericardial tamponade, surgical repair can be an option for restenosis or complete occlusion cases. We herein report a case of surgical repair with sutureless technique using the left atrial appendage (LAA) for complete occlusion of the left PV due to catheter ablation for atrial fibrillation (AF).

## Case presentation

**Fig. 1:**
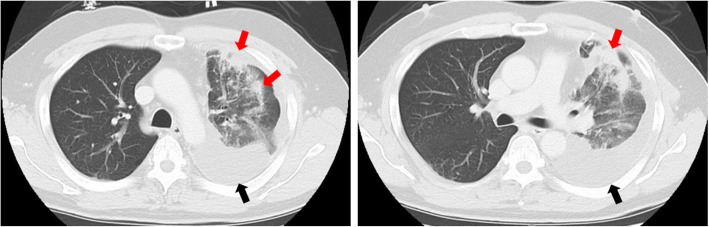
Preoperative CT of the lung. Multiple consolidation (red arrow) in the left lung and ipsilateral pleural effusion (black arrow)

**Fig. 2:**
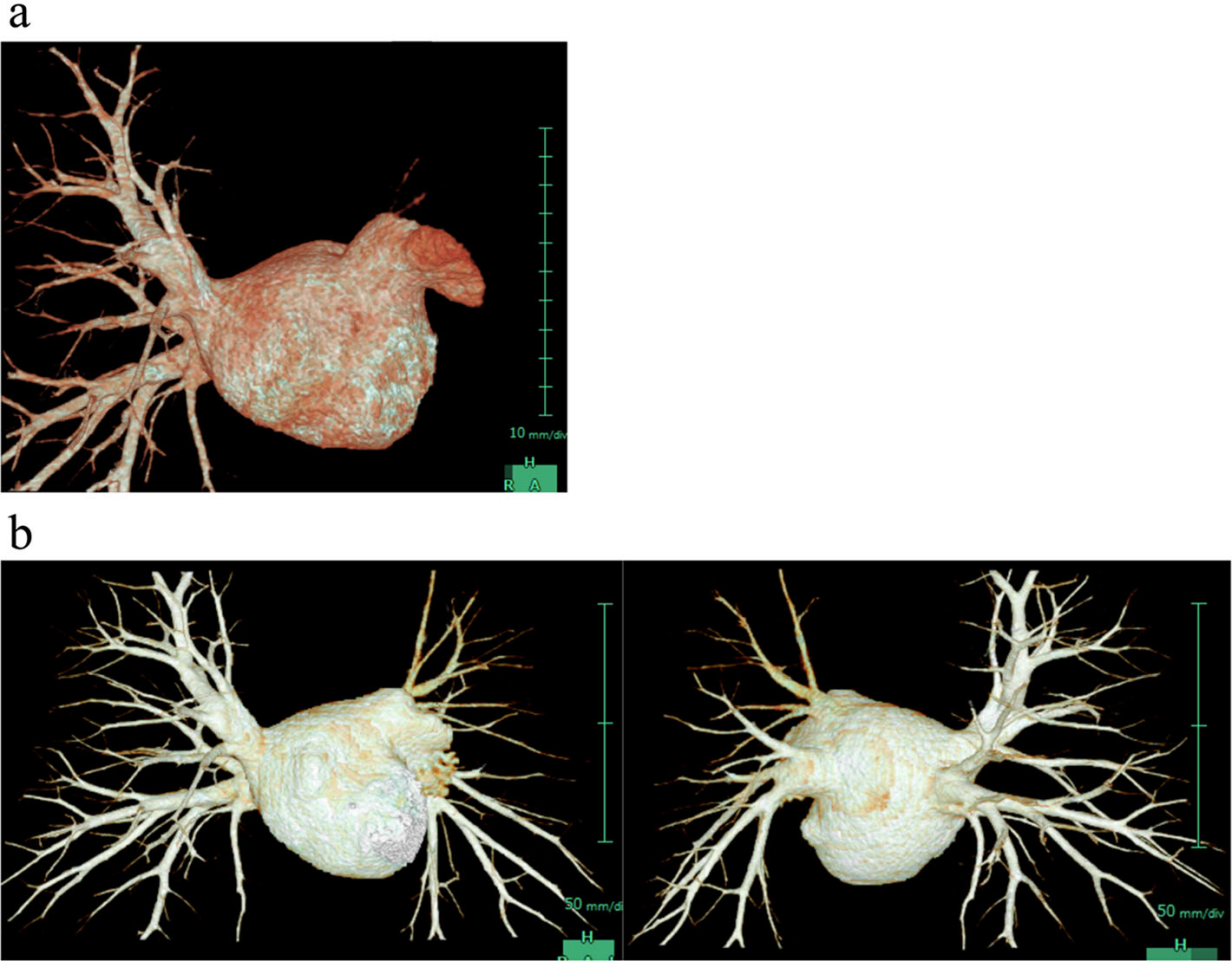
**a** Preoperative CT: The left PV was completely occluded at the connfluence. **b** Postoperative CT: The upper and lower branches were relatively narrow but both patent

**Fig. 3:**
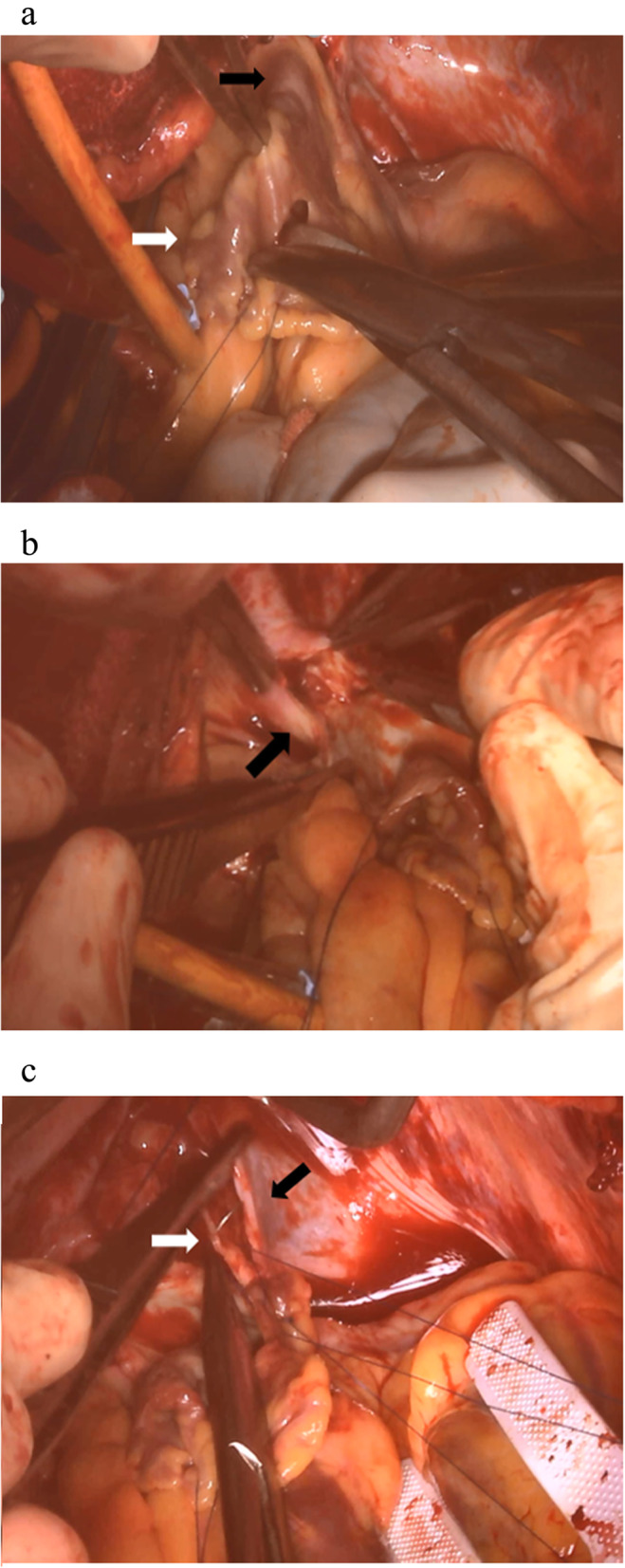
Intraoperative images. **a** The left atrium was incised from the apex of the LAA (white arrow). The black arrow showed the left upper PV. **b** The ostium of the left upper PV was completely occluded with fibrous scaring (black arrow). **c** The anastomosis line switched from the left atrium (white arrow) to the pericardium (black arrow)

**Fig. 4:**
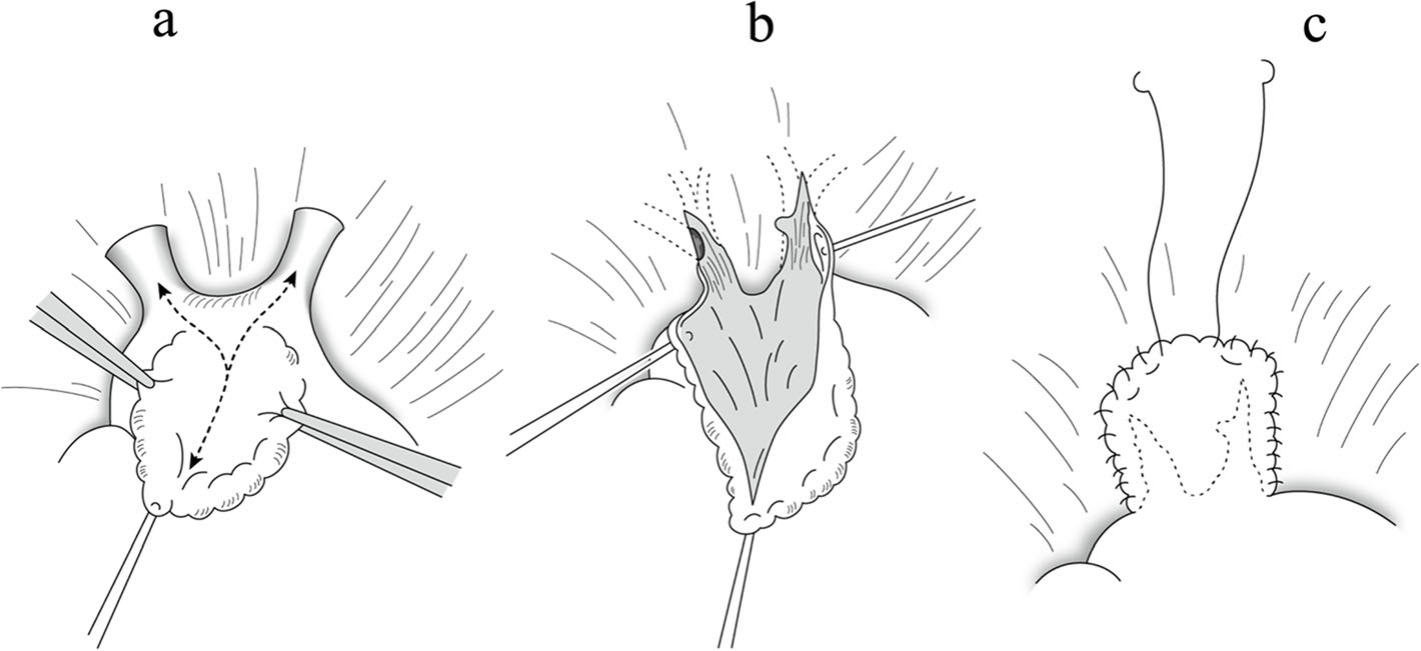
Schema of surgical procedures (illustration). **a** The left atrium was incised from the apex of the LAA to the confluence of the PV and proceeded separately to the upper and lower PVs. **b** The anterior walls of stenosed or occluded lesions were dissected as peripherally as possible. **c** The left atrium and PV were covered with incised LAA flap using 5-0 monofilament running suture (sutureless technique)

A 52-year-old male with AF underwent catheter ablation for two times. Eight months after the second maneuver for recurring AF, he presented with hemoptysis and exertional dyspnea. Computed tomography (CT) showed multiple consolidation in the left lung and ipsilateral pleural effusion (Fig. 1). Three-dimensional CT revealed severe stenosis of the left PV, and 2 months later, it was completely occluded (Fig. 2a). Since the symptoms grew more serious and transcatheter angioplasty seemed to be unfeasible due to little channels in ostia of PVs and has a certain risk of restenosis, surgical repair was planned. Under general anesthesia, cardiopulmonary bypass was initiated by aortobicaval cannulation via median sternotomy. A vent cannula was inserted into the left atrium through the right upper PV. After cross-clamping of the aorta and cardiac arrest with antegrade cardioplegia, the heart was manually retracted to the right. The left atrium was incised longitudinally from the apex of the LAA, and then the incision was separately extended towards the upper and lower PVs, respectively (Fig. 3a). The ostia of the PVs were completely occluded in the upper PV (Fig. 3b) and severely stenosed in the lower PV with fibrous scarring. The anterior wall of the stenotic or occluded lesions was dissected while the branches of the lower PV were relatively intact, and the upper PV was continuously stenosed to the peripheral branches. The incision was extended as peripherally as possible, but not to exceed the pericardium. The incised PVs and left atrium were covered with the incised LAA flap using a 5-0 monofilament running suture. The anastomosis line switched from the left atrium to the pericardium (Fig. 3c) and kept at least 5 mm away from the edge of the dissected PV walls to avoid direct suturing of venotomies. We also paid attention to the left phrenic nerve. The illustrated schema of the procedures is described in Fig. 4. These procedures were carried out under intermittent hypothermic circulatory arrest (28 °C) for prediction of the substantial bleeding from the collateral circulation such as the internal thoracic artery or bronchial artery. Maze procedure with cryoablation (Cryo ICE, AtriCure, OH, USA) was concurrently applied. After the surgery, the patient recovered sinus rhythm and was discharged without any complications. Enhanced CT demonstrated the patency of both left upper and lower PVs (Fig. 2b). Although the patient has maintained sinus rhythm afterwards, anticoagulant therapy was begun the following day after the surgery and has been proceeded with precautions against recurrence of AF and stenosis.

## Discussion

PV stenosis often develops as a congenital anatomical anomaly or anastomotic stenosis occurring after the repair of total anomalous PV connection, and several surgical repairs including endovenectomy, re-implantation of PV with direct anastomosis, or patchplasty have been described [2, 3]. However, restenosis of the PVs occurred due to proliferative neointimal hyperplasia in some cases, and direct suture of PV walls or patch materials was defined as a risk factor [4]. The sutureless technique, which requires no direct suture of PV walls, was initially described for those restenosis cases [4] and has been widely performed due to its good restenosis-free rate. This technique also reduces the distortion of the pulmonary venous structures by suturing on the unpliable pericardium, resulting in less local turbulence which may be another risk factor for restenosis [5]. Furthermore, especially in case of left PV stenosis, using the incised LAA as a flap reduces the risk of thromboembolism due to AF since the LAA is the most common site for cardiac thrombus. In addition, avoiding patch materials also reduces the risk of restenosis.

## Conclusion

The sutureless technique using the LAA for the left PV stenosis or occlusion after catheter ablation for AF is a significantly reasonable procedure in terms of reducing the risks of restenosis and thromboembolism. Since the long-term outcome of this technique is still unknown, careful follow-up is required.
